# Changes in Antimicrobial Resistance Patterns in Intensive Care Units Following the COVID-19 Pandemic: A 10-Year Retrospective Study from Türkiye

**DOI:** 10.3390/antibiotics15070636

**Published:** 2026-06-25

**Authors:** Ayşe Çapar, Derya Özyiğitoğlu, Şeyma Başlılar, Mürşide Efil Erdoğan, Beril Balak, Betül Nur Doğan, Öznur Hun Aktaş, Ebru Korkmaz

**Affiliations:** 1 Anaesthesiology and Intensive Care Medicine, Sultan Abdulhamid Han Training and Research Hospital, 34668 Istanbul, Türkiye; 2 Infectious Diseases and Clinical Microbiology, Sultan Abdulhamid Han Training and Research Hospital, 34668 Istanbul, Türkiye; deryamus@hotmail.com; 3 Chest Diseases, Sultan Abdulhamid Han Training and Research Hospital, 34668 Istanbul, Türkiye; seymabaslilar@yahoo.com (Ş.B.); oznur_hun@hotmail.com (Ö.H.A.); eebruukorkmaz@gmail.com (E.K.); 4 Pulmonary Intensive Care Unit, Sultan Abdulhamid Han Training and Research Hospital, 34668 Istanbul, Türkiye; mursideefil@gmail.com; 5 General Practice, Chelsea and Westminster Hospital, London SW10 9NH, UK; berilbalak@gmail.com; 6 Cardiothoracic Surgery Intensive Care Unit, Imperial College Healthcare NHS Trust, Hammersmith Hospital, London W12 0HS, UK; betuldogannur@gmail.com

**Keywords:** COVID-19 pandemic, antimicrobial resistance, intensive care unit, Gram-negative bacteria, *Candida auris*

## Abstract

Background: The coronavirus disease 2019 (COVID-19) pandemic coincided with substantial changes in healthcare delivery and antimicrobial resistance (AMR) patterns worldwide, particularly in intensive care units (ICUs), where invasive procedures and broad-spectrum antibiotics are commonly used. Data from Türkiye remains limited. Methods: This retrospective observational study evaluated bacterial and fungal isolates from adult ICU patients at a tertiary hospital from 2016 to 2025. Microorganisms were identified, and antimicrobial susceptibility testing was performed using standardized methods. Resistance patterns were compared between the pre-pandemic (January 2016–February 2020) and post-pandemic (March 2020–May 2025) periods. Results: A total of 2666 patients and 5433 isolates were analyzed. Gram-negative pathogens showed marked increases in resistance: carbapenem and colistin resistance in *Klebsiella pneumoniae* were significantly higher in the post-pandemic period (69.6% vs. 44.4% and 60.5% vs. 22.5%, respectively; *p* < 0.001). Resistance rates to multiple antimicrobial agents also increased in *Acinetobacter baumannii* and *Pseudomonas aeruginosa* (*p* < 0.05). Among Gram-positive bacteria, vancomycin-resistant *Enterococcus faecium* increased from 10% to 47.1%. *Candida auris* emerged only in the post-pandemic period, showing high resistance to fluconazole (75%) and amphotericin B (36.7%). Conclusions: Significant differences in AMR patterns were observed between the pre- and post-pandemic periods in this ICU population. Higher resistance rates were observed among several clinically important bacterial pathogens, and *Candida auris* emerged exclusively during the post-pandemic period. Given the study’s observational design, these findings should be interpreted as temporal associations rather than evidence of a causal effect of the COVID-19 pandemic. Continued antimicrobial stewardship and infection-control measures remain essential to address the growing burden of AMR.

## 1. Introduction

Coronavirus disease 2019 (COVID-19) is caused by severe acute respiratory syndrome coronavirus 2 (SARS-CoV-2). First identified in Wuhan, China, COVID-19 was declared a pandemic by the World Health Organization (WHO) on 11 March 2020. Since then, more than 777 million confirmed cases and over 7.1 million deaths have been reported worldwide, making COVID-19 one of the most significant global public health emergencies in recent decades [1].

Another primary public health concern is the rising problem of antimicrobial resistance (AMR). Independent of COVID-19, AMR remains among the top ten global health threats and is expected to result in up to 10 million deaths by 2050. [2,3]. Currently, about 700,000 patients die each year because of AMR [4]. However, several studies have shown that the prevalence of AMR has increased further during the COVID-19 pandemic. In particular, the frequent use of invasive devices in intensive care settings—such as mechanical ventilation (MV), renal replacement therapies (RRT), and central venous catheters—has been considered to significantly contribute to this resistance [5,6]. Thus, COVID-19 and AMR are two significant public health challenges that overlap [7].

Assessing AMR rates in Türkiye is crucial not only for understanding the impact of the COVID-19 pandemic but also for highlighting the long-term effects of antibiotic use and infection control measures. However, the pandemic served as an additional factor accelerating resistance, mainly through widespread antibiotic use and increased pressure on healthcare systems [8]. Although several years have passed since the pandemic started, it remains unclear whether this effect persists. Therefore, comparing resistance rates of major pathogens collected before and after the COVID-19 period is essential for understanding the current epidemiological situation in Türkiye and guiding future antimicrobial stewardship efforts.

## 2. Results

### 2.1. Descriptive Data

A total of 2666 patients and 5433 clinical specimens were included in the study. Of these, 948 patients (35.6%) and 1935 (35.6%) specimens were from the pre-pandemic period, and 1718 (64.4%) and 3498 (64.4%) specimens were from the post-pandemic period. The distribution of sample sources was as follows: 36.4% from respiratory secretions such as sputum, bronchoalveolar lavage, or tracheal aspirates (*n* = 683 vs. 1297), 24.3% from blood (*n* = 544 vs. 774), 33.1% from urine (*n* = 650 vs. 1146), 2.1% from rectal colonization samples (*n* = 1 vs. 113), and 4.1% from other body fluids (*n* = 57 vs. 168).

The average age of patients was 72.5 ± 14.9 years, and similar between the two study periods (*p* = 0.583). Mortality rates were 67.8% in the post-pandemic period and 62.1% in the pre-pandemic period (*p* = 0.003). Analysis of comorbidities revealed significantly higher rates in the post-pandemic group, including hypertension (HT) (53.9% vs. 36.8%; *p* < 0.001), coronary artery disease (CAD) (19.8% vs. 10.4%; *p* < 0.001), chronic obstructive pulmonary disease (COPD) (20% vs. 15.4%; *p* = 0.004), arrhythmia (12.2% vs. 5.4%; *p* < 0.001), congestive heart failure (CHF) (18.6% vs. 15.4%; *p* = 0.039), and malignancy (30% vs. 23.7%; *p* < 0.001) (Table 1).

### 2.2. Gram-Negative Bacteria

#### 2.2.1. *Acinetobacter baumannii*

**Figure 1:**
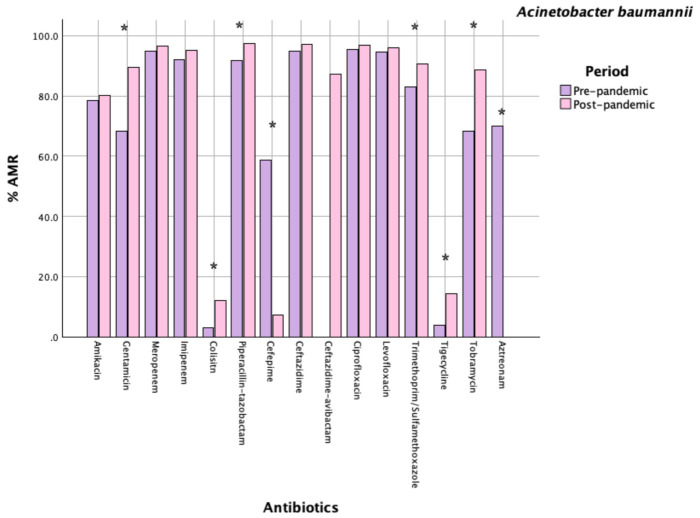
AMR patterns of *Acinetobacter baumannii* during the pre- and post-pandemic periods (* *p* < 0.05). AMR: Antimicrobial resistance.

A total of 1012 *Acinetobacter baumannii* isolates were identified, with 380 collected before the pandemic and 632 afterward. Most isolates came from respiratory specimens (59.5% vs. 68.5%). Meropenem resistance, which was 94.9% prior to the pandemic, increased to 96.6% afterward; however, this change was not statistically significant (*p* = 0.205). In contrast, colistin resistance rose significantly from 3% to 11.9% (*p* < 0.001). Resistance rates to trimethoprim-sulfamethoxazole and tigecycline also increased notably (*p* < 0.001). Furthermore, there was a significant rise in resistance to gentamicin and tobramycin during the post-pandemic period (*p* < 0.001) (Table 2, Figure 1).

#### 2.2.2. *Klebsiella pneumoniae*

**Figure 2:**
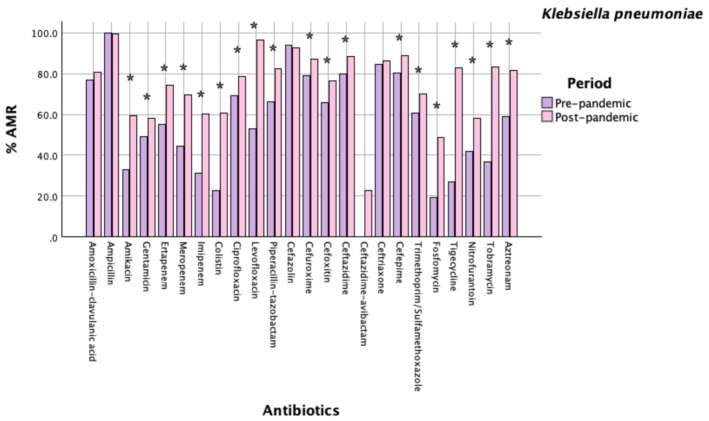
AMR patterns of *Klebsiella pneumoniae* during the pre- and post-pandemic periods (* *p* < 0.05). AMR: Antimicrobial resistance.

A total of 1257 *Klebsiella pneumoniae* isolates were identified, with 405 recovered during the pre-pandemic period and 852 during the post-pandemic period. Most isolates were obtained from respiratory specimens (37.5% vs. 44.7%). In the post-pandemic period, resistance rates to all carbapenems increased significantly (meropenem: 69.6% vs. 44.4%, *p* < 0.001; ertapenem: 74.3% vs. 55%, *p* < 0.001; imipenem: 60.2% vs. 31.1%, *p* < 0.001). Colistin resistance rose sharply from 22.5% to 60.5% (*p* < 0.001). Additionally, resistance increased significantly against amikacin, gentamicin, piperacillin-tazobactam, cephalosporins, fluoroquinolones, trimethoprim-sulfamethoxazole, fosfomycin, and tigecycline (*p* < 0.05) (Table 2, Figure 2).

#### 2.2.3. *Pseudomonas aeruginosa*

**Figure 3:**
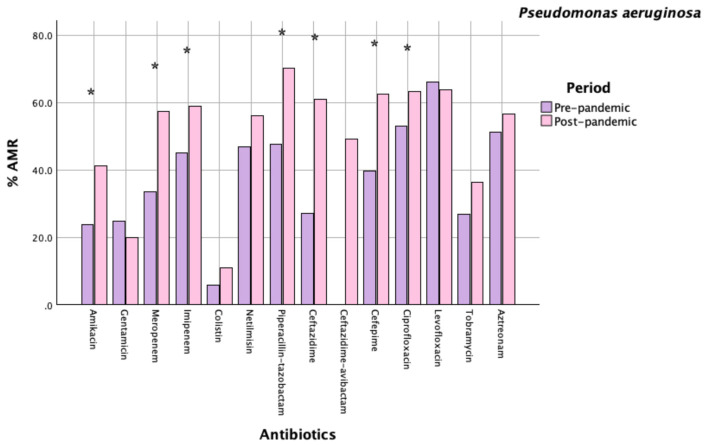
AMR patterns of *Pseudomonas aeruginosa* during the pre- and post-pandemic periods (* *p* < 0.05). AMR: Antimicrobial resistance.

A total of 648 isolates of *Pseudomonas aeruginosa* were identified, with 236 detected during the pre-pandemic period and 412 during the post-pandemic period. The majority of isolates came from respiratory samples (54.7% vs. 60.4%). Meropenem resistance increased significantly from 33.7% in the pre-pandemic period to 57.3% post-pandemic (*p* < 0.001). Significant increases were also seen in resistance to amikacin (41.3% vs. 23.8%; *p* < 0.001), piperacillin-tazobactam (70.3% vs. 47.7%; *p* < 0.001), and ceftazidime (61.1% vs. 27.3%; *p* < 0.001). Resistance to ciprofloxacin also showed a significant rise (*p* = 0.013) (Table 3, Figure 3).

#### 2.2.4. *Escherichia coli*

A total of 946 *Escherichia coli* isolates were identified, with 371 detected during the pre-pandemic period and 575 during the post-pandemic period. Most isolates were obtained from urine samples (60.6% vs. 63.1%). Resistance to amoxicillin-clavulanate decreased from 66.2% in the pre-pandemic period to 52.4% post-pandemic (*p* < 0.001). However, significant increases were observed in resistance to amikacin (11.4% vs. 5.5%; *p* = 0.01), levofloxacin (93.3% vs. 69%; *p* = 0.003), and tigecycline (9% vs. 2.1%; *p* = 0.008) (Table 3).

#### 2.2.5. *Proteus mirabilis*

A total of 125 isolates of *Proteus mirabilis* were identified, with 42 detected during the pre-pandemic period and 83 cases in the post-pandemic period. Most isolates were obtained from urine samples (57.1% vs. 56.6%). Meropenem resistance increased from 7.1% pre-pandemic to 24.5% post-pandemic, reaching borderline statistical significance (*p* = 0.055). In contrast, fluoroquinolone resistance declined, with ciprofloxacin resistance dropping from 79.5% to 56.6% (*p* = 0.014). Although not statistically significant, high colistin resistance was observed in both periods (95.5% vs. 100%, *p* = 1) (Table S1).

#### 2.2.6. *Stenotrophomonas maltophilia* and *Serratia marcescens*

A total of 112 isolates of *Stenotrophomonas maltophilia* (23 pre-pandemic vs. 89 post-pandemic) and 57 isolates of *Serratia marcescens* (28 pre-pandemic vs. 29 post-pandemic) were identified. Both pathogens were mainly isolated from respiratory specimens (60.9% vs. 51.7% for *Stenotrophomonas maltophilia*; 46.4% vs. 58.6% for *Serratia marcescens*). No significant changes in resistance rates were observed over time. In *Serratia marcescens*, overall AMR remained consistently high, with notably high colistin resistance in both the pre- and post-pandemic periods (87.6% vs. 86.7%, *p* = 1) (Table S1).

### 2.3. Gram-Positive Bacteria

#### 2.3.1. *Enterococcus faecalis*

**Figure 4:**
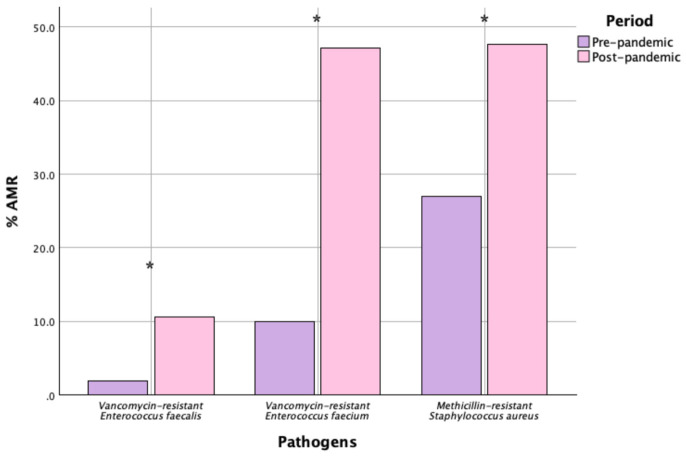
AMR patterns of Gram-positive bacteria during the pre- and post-pandemic periods (* *p* < 0.05). AMR: Antimicrobial resistance.

A total of 401 isolates of *Enterococcus faecalis* were identified, with 183 detected during the pre-pandemic period and 218 during the post-pandemic period. Most isolates were obtained from urine samples (55.7% vs. 69.7%). While no rectal colonization was observed in the pre-pandemic period, this rate rose to 4.1% afterward. Resistance to ampicillin decreased significantly from 14.1% to 4.8% (*p* = 0.002). Conversely, vancomycin resistance increased from 1.9% to 10.6% (*p* = 0.008), and there was also a significant rise in tigecycline resistance (*p* < 0.001) (Table 4, Figure 4).

#### 2.3.2. *Enterococcus faecium*

A total of 392 *Enterococcus faecium* isolates were identified, with 95 recovered during the pre-pandemic period and 297 during the post-pandemic period. Most isolates were obtained from urine specimens (55.8% compared to 43.1%). Rectal colonization was detected in 1.1% of cases pre-pandemic, increasing significantly to 35.0% post-pandemic. Vancomycin resistance increased significantly from 10.0% in the pre-pandemic period to 47.1% post-pandemic (*p* < 0.001). Significant increases in resistance to teicoplanin and tigecycline were also observed (*p* < 0.001) (Table 4, Figure 4).

#### 2.3.3. *Staphylococcus aureus*

A total of 212 isolates of *Staphylococcus aureus* were identified, with 86 detected during the pre-pandemic period and 126 during the post-pandemic period. Although *Staphylococcus aureus* was more frequently isolated from respiratory specimens in the pre-pandemic period (46.5%), it was mainly recovered from blood samples after the pandemic (54.0%). Oxacillin [*Methicillin-resistant Staphylococcus aureus* (*MRSA*)] resistance increased significantly from 27.0% to 47.6% (*p* = 0.029). Resistance rates to clindamycin, erythromycin, and penicillin remained unchanged (*p* > 0.05) (Table 4, Figure 4).

### 2.4. Fungal Infections

No statistically significant differences in antifungal resistance rates were observed between the pre- and post-pandemic periods for *Candida parapsilosis*, *Candida tropicalis*, and *Candida glabrata* isolates (*p* > 0.05, for both). For *Candida albicans*, a statistically significant decrease was observed only for caspofungin resistance (10% vs. 0%, *p* = 0.041), whereas resistance rates for the remaining antifungal agents were similar between the two periods (*p* > 0.05 for both). 

*Candida auris* isolates were identified only during the post-pandemic period and showed resistance rates of 36.7% to amphotericin B, 75.0% to fluconazole, 3.1% to caspofungin, and 3.0% to micafungin.

*Candida auris* was not detected during the pre-pandemic period and was first identified in 2020. The number of isolates increased over time, with 2 cases in 2020, 2 in 2021, 4 in 2022, 5 in 2023, 15 in 2024, and 6 cases identified from January through May 2025.

### 2.5. Longitudinal Trends in AMR Across the Study Period

**Figure 5:**
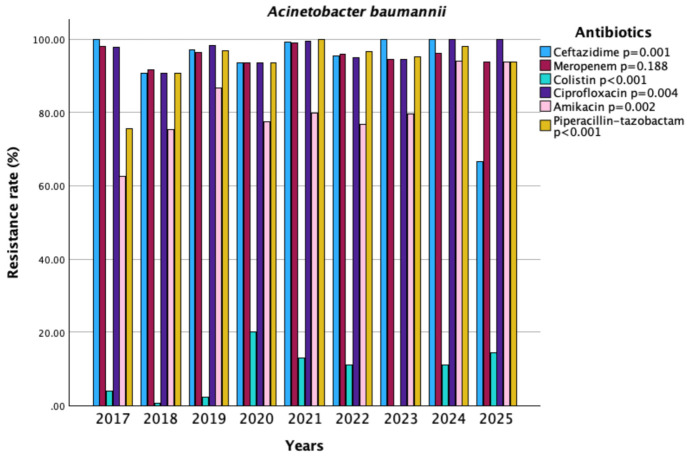
Trends in AMR patterns of *Acinetobacter baumannii* isolates over time.

Year-by-year analyses demonstrated temporal changes in AMR rates among major ICU pathogens during the study period. In *Acinetobacter baumannii*, significant increases were identified in resistance to ceftazidime (*p* = 0.001), colistin (*p* < 0.001), ciprofloxacin (*p* = 0.004), amikacin (*p* = 0.002), and piperacillin–tazobactam (*p* < 0.001), whereas meropenem resistance did not show a statistically significant temporal change (*p* = 0.188) (Figure 5).

**Figure 6:**
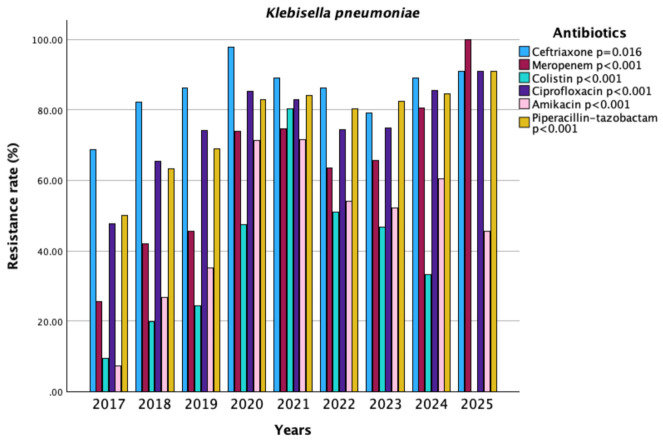
Trends in AMR patterns of *Klebsiella pneumoniae* isolates over time.

In *Klebsiella pneumoniae*, significant temporal increases were observed in resistance to ceftriaxone (*p* = 0.016), meropenem (*p* < 0.001), colistin (*p* < 0.001), ciprofloxacin (*p* < 0.001), amikacin (*p* < 0.001), and piperacillin–tazobactam (*p* < 0.001) (Figure 6).

**Figure 7:**
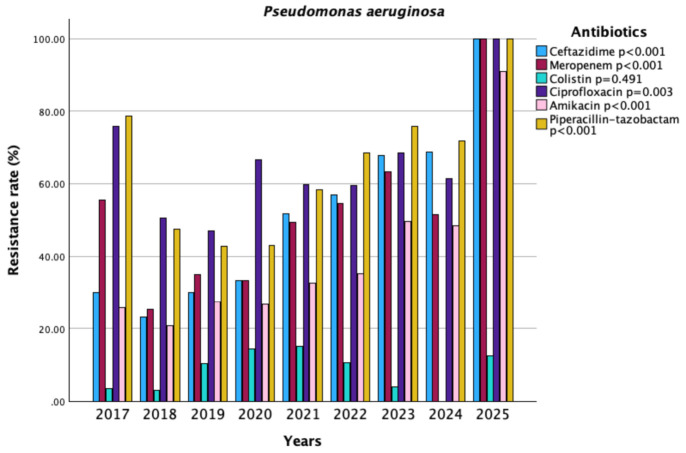
Trends in AMR patterns of *Pseudomonas aeruginosa* isolates over time.

Similarly, *Pseudomonas aeruginosa* demonstrated significant temporal increases in resistance to ceftazidime (*p* < 0.001), meropenem (*p* < 0.001), ciprofloxacin (*p* = 0.003), amikacin (*p* < 0.001), and piperacillin–tazobactam (*p* < 0.001). No statistically significant temporal change was observed for colistin resistance (*p* = 0.491) (Figure 7).

**Figure 8:**
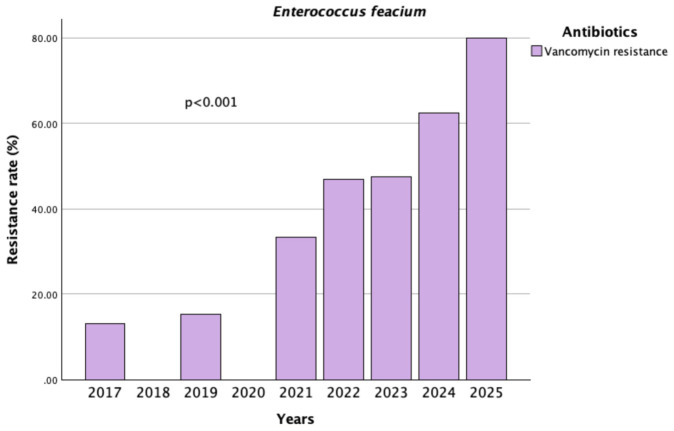
Temporal trends in vancomycin resistance rates of *Enterococcus faecium* over the years.

Among Gram-positive pathogens, vancomycin resistance in *Enterococcus faecium* increased significantly over the study period (*p* < 0.001) (Figure 8).

Visual inspection of annual resistance trends suggested a more pronounced increase in resistance rates after 2020, particularly for *Klebsiella pneumoniae:* meropenem, colistin, piperacillin-tazobactam, and amikacin resistance; for *Pseudomonas aeruginosa*: ceftazidime, meropenem, and piperacillin-tazobactam resistance; and for *Enterococcus faecium*: vancomycin resistance.

## 3. Discussion

In this retrospective, single-center intensive care unit (ICU) study spanning a 10-year period, significant differences in AMR patterns were observed between the pre- and post-pandemic periods, particularly among Gram-negative pathogens. Resistance rates were higher in the post-pandemic period for *Klebsiella pneumoniae*, *Acinetobacter baumannii*, and *Pseudomonas aeruginosa* across several commonly used antimicrobial agents, including meropenem, colistin, ceftazidime, ciprofloxacin, and piperacillin–tazobactam. Among Gram-positive bacteria, vancomycin resistance in *Enterococcus faecium* and *MRSA* rates were also significantly higher in the post-pandemic period. In addition, *Candida auris* isolates were identified exclusively during the post-pandemic period and exhibited high rates of resistance to fluconazole and amphotericin B.

COVID-19 has been a major public health challenge that coincided with substantial changes in healthcare systems worldwide. AMR, already recognized as one of the leading global health threats before the pandemic, remained a major concern throughout this period. Previous studies have suggested a potential association between the COVID-19 pandemic and changes in AMR patterns; however, this relationship is complex and likely influenced by multiple factors, including changes in healthcare delivery, patient populations, infection control practices, and antimicrobial prescribing patterns [9,10,11]. Although COVID-19 and AMR have been described as parallel and interacting public health challenges [11], the specific mechanisms underlying observed changes in resistance patterns remain incompletely understood [4]. Furthermore, because detailed antimicrobial consumption data were unavailable in the present study, the potential contribution of antibiotic use to the observed resistance patterns could not be directly assessed.

Carbapenem resistance among *Enterobacterales* poses a major global health threat. Because these antibiotics are often considered last-resort treatment options, resistance substantially limits therapeutic choices and is associated with adverse clinical outcomes [12]. *Klebsiella pneumoniae*, a member of the *Enterobacterales* family, is a well-known nosocomial pathogen capable of acquiring resistance to multiple antimicrobial agents. Because of its multidrug-resistant profile, it is often referred to as a “superbug.” In a six-year study, Hogea et al. reported rising resistance rates, including carbapenem resistance, among *Klebsiella pneumoniae* isolates [12]. Similarly, in our cohort, carbapenem resistance rates were significantly higher in the post-pandemic period than in the pre-pandemic period.

The situation is similar for *Acinetobacter baumannii*. In our study, although the increase in carbapenem resistance over the years did not reach statistical significance, resistance rates consistently exceeded 90%. *Acinetobacter baumannii* was designated by the WHO in 2017 as a critical priority pathogen posing a significant threat to human health. A global study reported more than one million *Acinetobacter baumannii* infections annually. Clinically, this pathogen most often causes ventilator-associated pneumonia and bloodstream infections. Its ability to persist on surfaces and survive in dry environments makes *Acinetobacter baumannii* a formidable cause of hospital-acquired infections. Notably, bacteria were found on the hands of healthcare workers caring for infected patients in about 30% of cases, highlighting an important route of transmission. Gram-negative bacteria can survive on hands for several minutes up to several hours. Additionally, the resistance of *Acinetobacter* species to common disinfectants may help it spread further within healthcare facilities [13,14].

*Pseudomonas aeruginosa* is a bacterium capable of surviving in moist environments and has a remarkable ability to acquire AMR. It is a common cause of ventilator-associated pneumonia and bloodstream infections. Previous studies have reported high rates of fluoroquinolone resistance among *Pseudomonas aeruginosa* isolates, which may be related to the widespread use of these agents in clinical practice. Indeed, *Pseudomonas aeruginosa* can develop resistance to all β-lactam antibiotics, and carbapenem resistance rates of up to 49.4% have been reported [15]. In our study, resistance rates to carbapenems and fluoroquinolones were significantly higher in the post-pandemic period than in the pre-pandemic period. Ceftazidime-avibactam, a third-generation cephalosporin combined with a β-lactamase inhibitor designed to target β-lactamases in *Pseudomonas aeruginosa*, has generally been reported worldwide with resistance rates below 10% [16]. However, in our cohort, the resistance rate was as high as 49.1%, indicating a substantial burden of resistance to this agent in our ICU population.

Another Gram-negative pathogen with higher carbapenem resistance rates observed during the study period was *Proteus mirabilis*. Along with other Gram-negative bacteria, it is increasingly recognized as a common hospital-acquired pathogen. Over the past twenty years, the incidence of *Proteus* infections has risen, with reported rates of approximately 4.4%, similar to the 4.6% rate observed in our cohort. Traditionally, β-lactam antibiotics were considered effective against this pathogen because it was not known to produce β-lactamases. However, over the past two decades, *Proteus mirabilis* isolates harboring multiple acquired resistance genes have increasingly been reported, complicating treatment. *Proteus mirabilis* can also form biofilms, which enhance its persistence and pathogenic potential. Urinary tract infections caused by this pathogen have been associated with increased mortality [17]. In our study, meropenem resistance rates were higher in the post-pandemic period than in the pre-pandemic period (24.5% vs. 7.1%), but this difference did not reach statistical significance (*p* = 0.055).

Colistin, also known as polymyxin E, is considered a last-resort antimicrobial for treating multidrug-resistant Gram-negative bacterial infections. Because therapeutic alternatives are often limited, the emergence of colistin resistance is a major clinical concern. One mechanism of acquired resistance involves the mcr-1 gene, which has been identified in various bacterial species and may spread through horizontal gene transfer. Bacteria carrying this gene have also been detected in urban wastewater, highlighting their potential public health implications [9]. In our study, colistin resistance rates were significantly higher in the post-pandemic period for both *Acinetobacter baumannii* and *Klebsiella pneumoniae* isolates, with rates exceeding 60% in *Klebsiella pneumoniae*. In addition, *Klebsiella pneumoniae* is known to form biofilms, a trait that may contribute to antimicrobial tolerance and persistence [18]. By contrast, the high colistin resistance rates observed in *Proteus mirabilis* and *Serratia marcescens* should be interpreted in the context of their intrinsic polymyxin resistance, a well-known microbiological characteristic of these species. Therefore, the resistance patterns observed in these organisms do not necessarily indicate newly acquired colistin resistance.

Compared with Gram-negative organisms, Gram-positive bacteria were less frequently isolated in our cohort. The most commonly identified pathogens were *Staphylococcus aureus*, *Enterococcus faecium*, and *Enterococcus faecalis*. The WHO has recognized *Staphylococcus aureus* as a priority pathogen threatening human health. Although approximately 30% of individuals are asymptomatic carriers of *Staphylococcus aureus* on the skin and mucosal surfaces, the organism can cause severe infections because it forms biofilms on both biotic and abiotic surfaces. Among *Staphylococcus aureus* isolates, *MRSA* remains a major public health concern, with reported prevalence rates ranging from 20% to 80% worldwide [19]. In our cohort, *MRSA* rates were significantly higher in the post-pandemic period, reaching 47.6%.

*Enterococci* are also important healthcare-associated pathogens capable of causing severe infections and have been associated with substantial morbidity and mortality among hospitalized patients [20]. Notably, *Enterococcus faecium* has been classified by the Centers for Disease Control and Prevention (CDC) as a serious threat due to vancomycin resistance [21]. Approximately 30% of enterococcal infections have been reported as vancomycin-resistant worldwide [22]. In a large surveillance study including Türkiye, vancomycin resistance rates ranged between 10% and 25% [23]. In our cohort, vancomycin resistance rates were higher during the post-pandemic period, reaching 47.1% in *Enterococcus faecium* and 10.6% in *Enterococcus faecalis*. An additional observation was a greater number of rectal screening isolates identified during the post-pandemic period, particularly for *Enterococcus faecium*. However, this finding should be interpreted cautiously, as changes in screening practices, surveillance intensity, and specimen distribution over time may have influenced detection rates. Because this retrospective study covered a 10-year period, detailed information on changes in rectal screening policies and infection-control surveillance protocols was not systematically available. Therefore, the potential contribution of surveillance practices to the increased detection of *Enterococcus* spp. colonization could not be fully assessed.

The emergence of fungal pathogens poses an additional challenge for critically ill patients. Previous studies have identified broad-spectrum antimicrobial exposure, invasive supportive therapies, and severe underlying illness as factors associated with an increased risk of invasive *Candida* infections. *Candida auris*, a multidrug-resistant fungal pathogen first identified in Japan in 2009, has since been reported in more than 40 countries worldwide and has attracted considerable attention because of its high levels of antifungal resistance. Reported resistance rates approach 90% for fluconazole and 30% for amphotericin B [24,25]. Owing to its clinical significance and outbreak potential, *Candida auris* has been classified as an “urgent threat” by the CDC and included on the WHO Critical Priority fungal pathogens list [25]. In our cohort, *Candida auris* isolates were identified exclusively during the post-pandemic period, with 34 isolates detected between 2020 and 2025. Resistance rates were 75% for fluconazole and 36.7% for amphotericin B, findings broadly consistent with international reports. Although the temporal emergence of *Candida auris* in our institution coincided with the post-pandemic period, the study’s observational design does not allow conclusions about a causal relationship with the COVID-19 pandemic. Nevertheless, these findings underscore the growing importance of *Candida auris* surveillance and infection-control measures in ICU settings.

In our institution, *Candida auris* was first identified in 2020 and showed a progressive increase during the study period, with 2 cases in 2020, 2 in 2021, 4 in 2022, 5 in 2023, 15 in 2024, and 6 cases recorded between January and May 2025. All *Candida auris* isolates included in this analysis were considered clinically significant infections and received antifungal treatment; therefore, they were not classified as colonization isolates. Although the marked increase observed in 2024 may suggest increased circulation of the organism within the ICU environment, formal outbreak investigations, transmission analyses, environmental surveillance data, and molecular epidemiological studies were unavailable due to the study’s retrospective design. Consequently, potential clustering events and transmission dynamics could not be assessed.

This study compared AMR patterns pre- and post-pandemic period at a large tertiary-care center in Türkiye and found higher rates of resistance in the post-pandemic period, particularly among Gram-negative pathogens. However, several important differences were also observed between the two study periods. Patients in the post-pandemic period had higher rates of comorbid conditions, including HT, CAD, COPD, arrhythmias, CHF, and malignancy, and overall mortality was higher. In addition, our institution substantially expanded ICU capacity during the pandemic, resulting in more admissions and a larger volume of microbiological samples. These changes may have influenced the observed resistance patterns independently of the pandemic itself. The observational design of the present study does not allow causal inferences regarding the relationship between the COVID-19 pandemic and AMR. Rather, the findings should be interpreted as temporal associations observed between the pre-pandemic and post-pandemic periods. Other factors, including changes in patient characteristics, healthcare utilization, infection-control practices, specimen distribution, and antimicrobial prescribing patterns, may also have contributed to the observed differences. Previous reports have highlighted widespread empirical antibiotic use during the COVID-19 era, with antibiotic exposure frequently exceeding the documented rates of bacterial or fungal co-infection [26]. Similarly, increased use of azithromycin, fluoroquinolones, and third-generation cephalosporins has been reported in Türkiye during the pandemic period [8]. However, because detailed antimicrobial consumption data—including defined daily doses (DDD), days of therapy (DOT), antimicrobial stewardship indicators, and prescribing records—were unavailable in the present study, the potential contribution of antibiotic use to the resistance patterns observed in our cohort could not be directly assessed. Therefore, no direct conclusions can be drawn about the relationship between antibiotic consumption and the observed resistance patterns.

Several changes to the healthcare system occurred during the pandemic and have been proposed as potential factors influencing AMR patterns. These include ICU expansion, increased patient admissions, greater reliance on invasive procedures, and challenges in maintaining infection-control practices during periods of heightened healthcare demand [27]. In addition, structural challenges within healthcare systems, such as staff shortages, workforce turnover, and limited palliative care resources, may affect antimicrobial stewardship efforts. Previous studies have also reported widespread corticosteroid use during the early phases of the COVID-19 pandemic, particularly before standardized treatment protocols were available [28,29]. Although corticosteroids later became an established component of treatment for selected patients with severe COVID-19, their use has been associated with an increased risk of secondary infections, including invasive fungal infections [30,31,32]. Nevertheless, the present study did not include patient-level data on corticosteroid exposure, antimicrobial use, infection-control practices, or stewardship interventions. Therefore, the potential contribution of these factors to the resistance patterns observed in our cohort cannot be directly assessed. Future multicenter studies incorporating detailed antimicrobial utilization and infection-control data are needed to better understand the mechanisms underlying changes in AMR patterns.

### Limitations

This study was conducted at a single center, which limits the generalizability of the findings. AMR patterns could not be thoroughly examined at the enzymatic level. Data on corticosteroid use, including which patients received them and at what doses, were not available in the patient records. Empirical antibiotic regimens varied across clinical departments, and no single agent was most commonly prescribed. Additionally, detailed analyses of AMR were not possible, as this was primarily a descriptive study. Finally, rates of secondary infections in the pre- and post-pandemic periods could not be assessed.

Furthermore, data on antimicrobial consumption, including DDD, DOT, antimicrobial stewardship metrics, and prescribing practices, were unavailable. Consequently, the relationship between antimicrobial use and resistance patterns could not be directly assessed.

In addition, numerous statistical comparisons were conducted across multiple pathogens and antimicrobial agents. No formal multiple-comparison adjustment was applied because the study was primarily exploratory and descriptive. Therefore, some statistically significant findings may represent type I errors and should be interpreted with caution, particularly for analyses with marginal *p*-values.

Another limitation of this study is the lack of molecular characterization of AMR mechanisms. Resistance patterns were evaluated using phenotypic susceptibility testing results, whereas resistance determinants such as carbapenemase genes, mcr-mediated colistin resistance genes, and vancomycin resistance genes (e.g., VanA and VanB) were not investigated. Therefore, the molecular mechanisms underlying the observed resistance patterns could not be determined.

The other is that antimicrobial susceptibility interpretations were based on Clinical and Laboratory Standards Institute (CLSI) criteria before 2023 and on European Committee on Antimicrobial Susceptibility Testing (EUCAST) criteria thereafter, reflecting routine laboratory practice during the study period. Because breakpoint definitions may differ between these guideline systems and may be revised over time, resistance estimates for certain antimicrobial agents could have been influenced by changes in interpretive criteria.

## 4. Materials and Methods

### 4.1. Study Design and Patient Selection

This retrospective observational study was conducted in the ICUs of İstanbul Sultan Abdulhamid Han Training and Research Hospital. Inclusion criteria were adult patients (≥18 years) admitted to our institution’s ICUs between January 2016 and May 2025 who had at least one bacterial or fungal isolate from a clinical specimen. Exclusion criteria included duplicate isolates of the same microorganism from the same specimen type in the same patient and cases with incomplete microbiological or demographic data.

### 4.2. Data Collection

Patients’ demographic data (age and sex), comorbidities [diabetes mellitus (DM), HT, CAD, COPD, malignancy, CHF, and arrhythmias], as well as clinical outcomes (mortality rates), were retrospectively gathered from the hospital’s electronic medical records. The analysis aimed to compare the study population across two distinct periods: the pre-COVID-19 pandemic period (January 2016–February 2020) and the post-pandemic period (March 2020–May 2025). In Türkiye, the first case was officially reported on 15 March 2020.

### 4.3. Microbiological Testing

Respiratory and non-respiratory specimens, including blood, urine, pleural fluid, tissue samples, and peritoneal fluid, were processed for bacterial identification using conventional culture methods and the VITEK 2 Compact automated identification system (bioMérieux, Marcy-l’Etoile, France). All samples were transported to the microbiology laboratory under appropriate conditions and processed according to specimen-specific standard microbiological procedures. Gram staining was performed for all appropriate specimens to evaluate cellular content and microbial morphology, while sputum samples were additionally assessed for adequacy prior to culture.

Blood samples were inoculated into blood culture bottles and incubated in automated blood culture systems; upon detection of microbial growth, Gram staining and subcultures onto solid media were performed. Urine specimens were cultured using quantitative methods on appropriate media, including sheep blood agar and MacConkey agar. Respiratory samples and other sterile body fluids were directly inoculated onto sheep blood agar, chocolate agar (incubated in 5% CO_2_), and MacConkey agar. All culture plates were incubated at 35–37 °C under aerobic conditions, with chocolate agar maintained in a 5% CO_2_ atmosphere. Cultures were evaluated after 18–24 h and extended up to 48 h when necessary.

Microorganism identification and antimicrobial susceptibility testing were performed in accordance with the standards of the CLSI-M100 and EUCAST guidelines. CLSI interpretive criteria were used from 2016 to 2022, whereas EUCAST criteria were adopted beginning in 2023. Antimicrobial susceptibility results were interpreted according to the standards routinely used in the corresponding study year. Because this retrospective study was based on routinely reported laboratory data, susceptibility results were not reclassified according to a single guideline system.

Candida species identification was performed using Sabouraud dextrose agar media and automated systems, while antifungal susceptibility testing followed CLSI M27 reference standards. Susceptibility to polymyxins was evaluated through the broth microdilution method. To reduce overrepresentation of repeated microbiological cultures, duplicate isolates from the same specimen type that identified the same microorganism in the same patient were excluded from the analysis. However, isolates recovered from different specimen sources (e.g., blood, respiratory, urine, or other samples) were considered independent microbiological events and were analyzed separately, even when the same microorganism was identified. Polymicrobial cultures were not analyzed separately; each eligible microorganism was included individually in the dataset. No predefined time-based deduplication interval was applied.

### 4.4. Statistical Analysis

Statistical analyses were performed using IBM SPSS Statistics for Mac, version 30.0 (IBM Corp., Armonk, NY, USA). The distribution of continuous variables was assessed with the Shapiro–Wilk and Kolmogorov–Smirnov test. Variables with a normal distribution are shown as mean ± standard deviation, while non-normally distributed variables are presented as median (minimum–maximum). Categorical variables are described as counts and percentages (%). Between-group comparisons for non-normal variables were conducted with the Mann–Whitney U test. Differences in categorical variables were evaluated using the Chi-square test or Fisher’s exact test, as appropriate. A *p*-value of <0.05 was considered statistically significant in all analyses.

For annual analyses, AMR rates were evaluated for each study year (2016–2025). Differences in resistance rates across study years were assessed using the Chi-square test or Fisher’s exact test, as appropriate. Annual resistance rates were presented graphically to visualize temporal patterns.

## 5. Conclusions

The present study demonstrated substantial differences in AMR patterns between the pre- and post-pandemic periods in a large tertiary-care ICU in Türkiye. Higher resistance rates were observed among several clinically important pathogens, particularly Gram-negative bacteria, during the post-pandemic period. In addition, *Candida auris* was identified exclusively during the post-pandemic period and showed high levels of antifungal resistance.

Given the study’s observational design, the findings should be interpreted as temporal associations rather than evidence of a causal effect of the COVID-19 pandemic on AMR. Multiple factors, including changes in patient characteristics, ICU capacity, healthcare utilization, infection-control practices, and antimicrobial prescribing patterns, may have contributed to the observed differences.

Nevertheless, the persistence of high resistance rates among major ICU pathogens highlights the ongoing challenge of AMR. Strengthening infection-control measures, promoting rational antimicrobial use, and maintaining robust national surveillance programs remain essential strategies for addressing this growing public health concern.

## Figures and Tables

**Table 1:** Comparison of clinical, demographic, and microbiological characteristics of patients in the pre- and post-pandemic periods.

Variables	Overall Mean ± sd or n (%) *n* = 2666	Pre-Pandemic Period Median (Min-Max) or n (%) *n* = 948	Post-Pandemic Period Median (Min-Max) or n (%) *n* = 1718	*p*-Value
Age	72.51 ± 14.92	75 (18–102)	75 (17–104)	0.583
Mortality	1754 (65.8)	589 (62.1)	1165 (67.8)	0.003
DM	822 (30.8)	284 (30)	538 (31.3)	0.467
HT	1275 (47.8)	349 (36.8)	926 (53.9)	<0.001
CAD	440 (16.5)	99 (10.4)	341 (19.8)	<0.001
COPD	489 (18.3)	146 (15.4)	343 (20)	0.004
Arrhythmias	265 (9.9)	56 (5.9)	209 (12.2)	<0.001
CHF	465 (17.4)	146 (15.4)	319 (18.6)	0.039
Malignancy	741 (27.8)	225 (23.7)	516 (30)	<0.001
**Pathogens**				<0.001
*Acinetobacter baumannii*	1012	380 (19.6)	632 (18.1)	
*Klebsiella pneumoniae*	1257	405 (20.9)	852 (24.4)	
*Pseudomonas aeruginosa*	648	236 (12.2)	412 (11.8)	
*Escherichia coli*	946	371 (19.2)	575 (16.4)	
*Staphylococcus aureus*	212	86 (4.4)	126 (3.6)	
*Enterococcus faecium*	392	95 (4.9)	297 (8.5)	
*Enterococcus faecalis*	401	183 (9.5)	218 (6.2)	
*Stenotrophomonas maltophilia*	112	23 (1.2)	89 (2.5)	
*Serratia marcescens*	57	28 (1.4)	29 (0.8)	
*Proteus mirabilis*	125	42 (2.2)	83 (2.4)	
*Candida albicans*	90	31 (1.6)	59 (1.7)	
*Candida parapsilosis*	86	33 (1.7)	53 (1.5)	
*Candida tropicalis*	42	18 (0.9)	24 (0.7)	
*Candida auris*	34	0 (0)	34 (1)	
*Candida glabrata*	19	4 (0.2)	15 (0.4)	
Number of the Samples	5433	1935 (35.6)	3498 (64.4)	

DM: Diabetes Mellitus, HT: Hypertension, CAD: Coronary Artery Diseases, COPD: Chronic Obstructive Pulmonary Disease, CHF: Congestive Heart Failure. Continuous variables were analyzed using the Mann–Whitney U test, while categorical variables were evaluated using the Chi-square test or Fisher’s exact test, as appropriate.

**Table 2:** AMR evaluation of *Acinetobacter baumannii* and *Klebsiella pneumoniae* in the pre- and post-pandemic periods.

Variables	*Acinetobacter baumannii*			*Klebsiella pneumoniae*		
	Pre-Pandemic Period (n %) *n* = 380	Post-Pandemic Period (n %) *n* = 632	*p*-Value	Pre-Pandemic Period (n %) *n* = 405	Post-Pandemic Period (n %) *n* = 852	*p*-Value
Amikacin	297 (78.6)	505 (80.3)	0.513	114 (32.9)	459 (59.3)	<0.001
Gentamicin	258 (68.4)	411 (89.5)	<0.001	196 (49.2)	372 (58.1)	0.005
Meropenem	357 (94.9)	593 (96.6)	0.205	148 (44.4)	509 (69.6)	<0.001
Imipenem	139 (92.1)	366 (95.3)	0.14	46 (31.1)	215 (60.2)	<0.001
Colistin	11 (3)	46 (11.9)	<0.001	62 (22.5)	219 (60.5)	<0.001
Piperacillin-tazobactam	331 (91.9)	608 (97.4)	<0.001	237 (66.4)	663 (82.5)	<0.001
Ceftazidime	334 (94.9)	451 (97.2)	0.087	259 (79.7)	694 (88.2)	<0.001
Ceftazidime-Avibactam	*	48 (87.3)	NA	*	74 (22.8)	NA
Ciprofloxacin	351 (95.5)	607 (97)	0.197	272 (69)	659 (78.6)	<0.001
Trimethoprim/Sulfamethoxazole	305 (82.9)	567 (90.6)	<0.001	240 (60.6)	584 (69.9)	0.001
Tigecycline	13 (3.7)	83 (14.2)	<0.001	74 (26.8)	131 (82.9)	<0.001
Samples			<0.001			<0.001
Sputum/BAL/DTA	226 (59.5)	433 (68.5)		152 (37.5)	381 (44.7)	
Blood	116 (30.5)	122 (19.3)		98 (24.2)	166 (19.5)	
Urine	27 (7.1)	47 (7.4)		150 (37)	251 (29.5)	
Others	11 (2.9)	30 (4.7)		5 (1.2)	54 (6.3)	

BAL: Bronchoalveolar lavage, DTA: Deep tracheal aspirate. Antimicrobial resistance rates in the pre- and post-pandemic periods were compared using the Chi-square or Fisher’s exact test, as appropriate. * Ceftazidime-avibactam was not in clinical use during the pre-pandemic period in Turkey. NA: Not applicable.

**Table 3:** AMR evaluation of *Pseudomonas aeruginosa* and *Escherichia coli* in the pre- and post-pandemic periods.

Variables	*Pseudomonas aeruginosa*			*Escherichia coli*		
	Pre-Pandemic Period (n %) *n* = 236	Post-Pandemic Period (n %) *n* = 412	*p*-Value	Pre-Pandemic Period (n %) *n* = 371	Post-Pandemic Period (n %) *n* = 575	*p*-Value
Amikacin	49 (23.8)	166 (41.3)	<0.001	14 (5.5)	46 (11.4)	0.01
Meropenem	68 (33.7)	221 (57.3)	<0.001	7 (3.2)	15 (4)	0.614
Imipenem	50 (45)	209 (58.9)	0.01	6 (4.1)	10 (5.7)	0.503
Colistin	10 (6)	18 (11)	0.1	3 (2.2)	1 (2)	1
Piperacillin-tazobactam	106 (47.7)	282 (70.3)	<0.001	57 (21.1)	118 (25.7)	0.161
Ceftazidime	62 (27.3)	248 (61.1)	<0.001	166 (67.8)	306 (67.8)	0.98
Ceftazidime-Avibactam	*	78 (49.1)	NA	*	3 (10.3)	NA
Ceftriaxone	–	–	NA	185 (75.5)	318 (68.4)	0.047
Cefepime	78 (39.8)	242 (62.5)	<0.001	105 (57.7)	194 (56.7)	0.831
Ciprofloxacin	120 (53.1)	253 (63.2)	0.013	226 (64.6)	356 (62.5)	0.518
Samples			0.011			0.07
Sputum/BAL/DTA	129 (54.7)	249 (60.4)		78 (21)	87 (15.1)	
Blood	49 (20.8)	48 (11.7)		59 (15.9)	101 (17.6)	
Urine	46 (19.5)	82 (19.9)		225 (60.6)	363 (63.1)	
Others	12 (5.1)	33 (8)		9 (2.4)	24 (4.2)	

BAL: Bronchoalveolar lavage, DTA: Deep tracheal aspirate. Antimicrobial resistance rates in the pre- and post-pandemic periods were compared using the Chi-square or Fisher’s exact test, as appropriate. * Ceftazidime-avibactam was not in clinical use during the pre-pandemic period in Turkey. NA: Not applicable. –: Not tested.

**Table 4:** AMR evaluation of *Enterococcus faecalis*, *Enterococcus faecium*, and *Staphylococcus aureus* in the pre- and post-pandemic periods.

Variables	*Enterococcus faecalis*			*Enterococcus faecium*			*Staphylococcus aureus*		
	Pre-Pandemic Period (n %) *n* = 183	Post-Pandemic Period (n %) *n* = 218	*p*-Value	Pre-Pandemic Period (n %) *n* = 95	Post-Pandemic Period (n %) *n* = 297	*p*-Value	Pre-Pandemic Period (n %) *n* = 86	Post-Pandemic Period (n %) *n* = 126	*p*-Value
Ampicillin	23 (14.1)	10 (4.8)	0.002	78 (91.8)	261 (94.6)	0.345	–	–	NA
Linezolid	3 (2.8)	4 (4.9)	0.469	3 (3.9)	3 (1.3)	0.17	0 (0)	1 (1.2)	1
Teicoplanin	2 (1.9)	10 (10.9)	0.007	7 (9)	118 (45.7)	<0.001	10 (15.6)	1 (1.3)	0.002
Vancomycin	2 (1.9)	11 (10.6)	0.008	9 (10)	130 (47.1)	<0.001	1 (1.6)	0 (0)	0.4
Tigecycline	1 (1.1)	23 (24.7)	<0.001	3 (4.3)	73 (37.8)	<0.001	–	–	NA
Oxacillin	–	–	NA	–	–	NA	10 (27)	50 (47.6)	0.029
Clindamycin	–	–	NA	–	–	NA	26 (33.8)	41 (35.3)	0.822
Erythromycin	–	–	NA	–	–	NA	7 (35)	26 (38.2)	0.793
Daptomycin	–	–	NA	–	–	NA	1 (6.7)	3 (6.4)	1
Samples			<0.001			<0001			0.025
Sputum/BAL/DTA	4 (2.2)	7 (3.2)		6 (6.3)	14 (4.7)		40 (46.5)	45 (35.7)	
Blood	72 (39.3)	49 (22.5)		34 (35.8)	45 (15.2)		29 (33.7)	68 (54)	
Urine	102 (55.7)	152 (69.7)		53 (55.8)	128 (43.1)		11 (12.8)	8 (6.3)	
Rectal	0 (0)	9 (4.1)		1 (1.1)	104 (35)		–	–	
Others	5 (2.7)	1 (0.5)		1 (1.1)	6 (2)		6 (7)	5 (4)	

BAL: Bronchoalveolar lavage, DTA: Deep tracheal aspirate. Antimicrobial resistance rates in the pre- and post-pandemic periods were compared using the Chi-square or Fisher’s exact test, as appropriate. NA: Not applicable, –: Not tested.

## Data Availability

The datasets used and/or analyzed during the current study are available from the corresponding author on reasonable request. The data are not publicly available due to ethical restrictions and the need to protect patient privacy.

## Supplementary Materials

The following supporting information can be downloaded at: https://www.mdpi.com/article/10.3390/antibiotics15070636/s1, Table S1: AMR evaluation of *Proteus mirabilis*, *Stenotrophomonas maltophilia*, and *Serratia marcescens* in the pre- and post-pandemic periods.

## Abbreviations

COVID-19	Coronavirus Disease-2019
SARS-CoV-2	Severe Acute Respiratory Syndrome Coronavirus 2
WHO	World Health Organization
AMR	Antimicrobial Resistance
MV	Mechanical Ventilation
RRT	Renal Replacement Therapies
HT	Hypertension
CAD	Coronary Artery Disease
COPD	Chronic Obstructive Pulmonary Disease
CHF	Chronic Heart Failure
*MRSA*	*Methicillin-resistant Staphylococcus aureus*
ICU	Intensive Care Unit
CDC	Centers for Disease Control and Prevention
DDD	Defined Daily Doses
DOT	Days of Therapy
CLSI	Clinical and Laboratory Standards Institute
EUCAST	European Committee on Antimicrobial Susceptibility Testing
DM	Diabetes Mellitus
