# Supplementary material for: Changes in Antimicrobial Resistance Patterns in Intensive Care Units Following the COVID-19 Pandemic: A 10-Year Retrospective Study from Türkiye
Source: Antibiotics (Basel). 2026 Jun 25;15(7):636. doi: 10.3390/antibiotics15070636 (PMC13403379; doi:10.3390/antibiotics15070636)
Supplement: Supplementary file 1 [file antibiotics-15-00636-s001.zip › antibiotics-4369408-supplementary.pdf]

Table S1. AMR evaluation of *Proteus mirabilis, Stenotrophomonas maltophilia,* and *Serratia marcescens* in the pre- and post-pandemic periods.

Variables	*Proteus mirabilis*			*Stenotrophomonas maltophilia*			*Serratia marcescens*		
	**Pre-pandemic period (n %) n=42**	**Post-pandemic period (n %) n=83**	**p-value**	**Pre-pandemic period (n %) n=23**	**Post-pandemic period (n %) n=89**	**p-value**	**Pre-pandemic period (n %) n=28**	**Post-pandemic period (n %) n=29**	**p-value**
Meropenem	2 (7.1)	13 (24.5)	0.055	–	–	NA	2 (1.3)	1 (1.7)	0.568
Colistin	18 (100)	21 (95.5)	1	–	–	NA	13 (86.7)	14 (87.5)	1.00
Piperacillin-tazobactam	4 (14.3)	16 (24.6)	0.266	–	–	NA	2 (8.7)	4 (14.8)	0.674
Ceftazidime	12 (41.4)	29 (46)	0.677	1 (25)	13 (36.1)	1.00	3 (15)	4 (16.7)	1.00
Cefepime	10 (50)	21 (42)	0.543	–	–	NA	3 (16.7)	3 (15)	1.00
Ciprofloxacin	31 (79.5)	47 (56.6)	0.014	–	–	NA	2 (7.4)	3 (10.7)	1.00
Levofloxacin	2 (100)	6 (85.7)	1	0 (0)	4 (7.3)	1.00	–	–	NA
Trimethoprim/ Sulfamethoxazole	28 (63.8)	55 (66.3)	0.821	3 (14.3)	10 (14.1)	1.00	–	–	NA
**Samples**			0.112			0.29			0.827
Sputum/BAL/DTA	11 (26.2)	14 (16.9)		14 (60.9)	46 (51.7)		13 (46.4)	17 (58.6)	
Blood	2 (4.8)	16 (19.3)		5 (21.7)	36 (40.4)		6 (21.4)	5 (17.2)	
Urine	24 (57.1)	47 (56.6)		3 (13)	5 (5.6)		8 (16.8)	6 (20.7)	
Others	5 (11.9)	6 (7.2)		1 (4.3)	2 (2.2)		1 (3.6)	2 (3.5)	

BAL: Bronchoalveolar lavage, DTA: Deep tracheal aspirate. Antimicrobial resistance rates in the pre- and post-pandemic periods were compared using the Chi-square or Fisher's exact test, as appropriate. NA: Not applicable, – Not tested.
